# Bandgap Pressure Coefficient of a CH_3_NH_3_PbI_3_ Thin Film Perovskite

**DOI:** 10.1021/acs.jpclett.3c01258

**Published:** 2023-07-12

**Authors:** Agnieszka Pienia̧żek, Filip Dybała, Maciej P. Polak, Łukasz Przypis, Artur P. Herman, Jan Kopaczek, Robert Kudrawiec

**Affiliations:** † Department of Semiconductor Materials Engineering, Wroclaw University of Science and Technology, Wybrzeże Wyspiańskiego 27, 50-370 Wrocław, Poland; ‡ Materials Science and Engineering Department, University of Wisconsin—Madison, Madison, Wisconsin 53706, United States; § Saule Research Institute, Wroclaw Technology Park, 11 Dunska Street, Sigma Building, 54-130 Wrocław, Poland

## Abstract

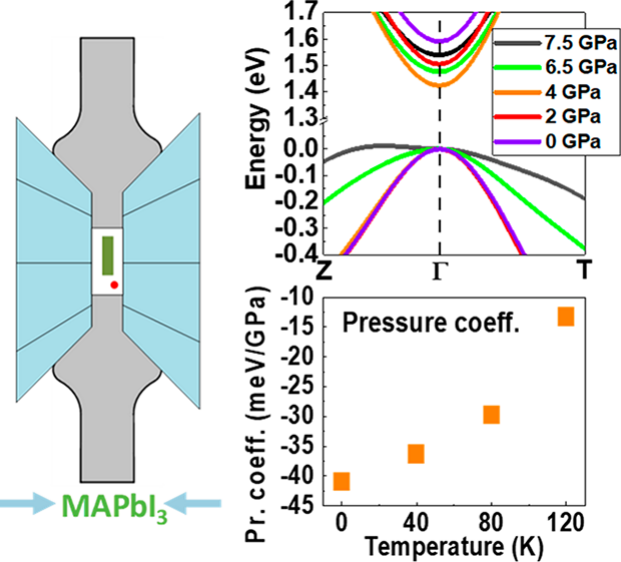

Recent scientific interest in examining the bandgap evolution of a MAPbI_3_ hybrid perovskite by applying hydrostatic pressure has mostly focused on a room-temperature tetragonal phase. In contrast, the pressure response of a low-temperature orthorhombic phase (OP) of MAPbI_3_ has not been explored and understood. In this research, we investigate for the first time how hydrostatic pressure alters the electronic landscape of the OP of MAPbI_3_. Pressure studies using photoluminescence combined with calculations within density functional theory at zero temperature allowed us to identify the main physical factors affecting the bandgap evolution of the OP of MAPbI_3_. The negative bandgap pressure coefficient was found to be strongly dependent on the temperature (α_120K_ = −13.3 ± 0.1 meV/GPa, α_80K_ = −29.8 ± 0.1 meV/GPa, and α_40K_ = −36.3 ± 0.1 meV/GPa). Such dependence is related to the changes in the Pb–I bond length and geometry in the unit cell as the atomic configuration approaches the phase transition as well as the increasing phonon contribution to octahedral tilting as the temperature increases.

Hybrid organic–inorganic perovskites have become one of the most exciting groups of photovoltaic (PV) materials. Facile processing combined with remarkable optoelectronic properties (such as strong light absorption, long carrier diffusion lengths, and long carrier lifetimes) make them promising compounds for use in high-efficiency PV technology.^1−4^ Methylammonium lead iodide (MAPbI_3_) with its relative ease and low cost of fabrication^1,5^ has emerged as a remarkable PV material with a power conversion efficiency at room temperature exceeding 21%.^6,7^ Moreover, due to its upscaling potential and cosmic radiation tolerance in the oxygen- and water-free space environment, MAPbI_3_ has been recently considered to be a promising candidate for space applications.^8^ To be qualified for space applications, PV modules must pass a standard thermal cycling test exposing them to temperature fluctuations over a wide range, including ultralow temperatures.^8−10^ This requirement can be challenging for MAPbI_3_ as it is poorly resistant to thermal decomposition and undergoes temperature-dependent phase transitions [above 330 K it exists in a cubic structure, between 330 and 160 K it is in the tetragonal phase (TP), and below 160 K it evolves into the OP^11−16^]. It has been found that the transition from the TP to the OP has a significant impact on the bandgap energy and, consequently, light absorption efficiency, which leads to changes in charge carrier generation, mobility, and extraction.^17,18^

Applying hydrostatic pressure is a way to tailor the bandgap of semiconducting materials by modifying the crystal structure, where changes in electronic behavior are induced by changes in interatomic and intermolecular distances without any introduction of additional chemical compounds. Moreover, combined with density functional theory (DFT) calculations, it helps to recognize structural factors affecting the electronic band structure with an atomic-level understanding. So far, all pressure-related studies have focused on the TP of MAPbI_3_ at room temperature.^4,19−27^ However, the pressure response of the optical and electronic properties of the low-temperature OP is of both fundamental and technological interest because, as mentioned above, MAPbI_3_ has the potential to power space missions. In this respect, we report the first attempt to investigate the electronic band structure evolution of the OP as well as its stability under hydrostatic pressure up to 6 GPa and determine the bandgap pressure coefficient in a low-temperature regime.

The MAPbI_3_ crystal is composed of corner-sharing [PbI_6_]^4–^ octahedra with the organic MA cations occupying the voids in the three-dimensional inorganic framework. The bandgap variation of the OP under hydrostatic pressure is determined by the contribution to the band structure of atomic orbitals near the Γ point in the Brillouin zone. The bandgap at the Γ point is dominated by the antibonding orbital composed of Pb 6s and I 5p orbitals as the valence band maximum and the antibonding orbital of the Pb 6p orbital as the conduction band minimum, while the MA cation contribution is negligible.^28,29^ The so-called inverted band structure in the perovskite, when compared to that of the conventional semiconductors, is related to the significant spin–orbit coupling due to the presence of heavy atoms, namely, lead and iodine.^30,31^

Intending to probe pressure-induced effects in the OP of MAPbI_3_, we inspected how the room-temperature tetragonal to low-temperature orthorhombic phase transition manifests in the optical property of this material and determined the temperature range in which the OP occurs by performing temperature-dependent photoluminescence (PL) studies under ambient pressure (1 atm). It is worth mentioning that pressure- and temperature-dependent PL values were measured for several MAPbI_3_ films with thicknesses of ∼250 nm. The results were reproducible; hence, we show only the representative ones. We find that the onset of the transition from the TP to the OP during the cooling cycle from 300 to 20 K is between 160 and 140 K, so the pressure response of the OP was studied at temperatures below 140 K. A detailed analysis of temperature-dependent PL results is presented in Note 1 and Figures S1–S3. Moreover, we performed an excitation power-dependent PL study of the MAPbI_3_ film to assess whether the observed PL response of the OP is related to the near-band-edge emission (instead of trap/defect states). Figure S4 shows PL spectra recorded at 120 K under ambient pressure and a continuous wave laser with the excitation power (*P*_exc_) ranging from 0.02 to 2 mW. The integrated PL intensity (*I*) is plotted as a function of *P*_exc_ on a log–log scale in the inset of Figure S4. Experimental points were fitted to the power law function of *I* ∝ *P*_exc_^Γ^ (solid line in the inset of Figure S4), where Γ depends on the type of radiative recombination process.^32^ For the considered sample, the fitting procedure yielded a Γ of 1.16 ± 0.01, which suggests that the PL emission arises mostly from the exciton recombination with no impact of defect-related recombination (for which Γ < 1). Furthermore, powder X-ray diffraction (XRD) measurement at room temperature was used to confirm the high crystal quality of the studied MAPbI_3_ film (Figure S5). Indeed, preferred orientations at 13.9° and 28.3° were observed and assigned to the (110) and (220) planes of the MAPbI_3_ tetragonal phase, respectively.^33^ Minor peaks of the (200), (211), (202), (310), (312), (224), and (330) planes are present at 2θ values of 19.8°, 23.2°, 24.2°, 31.6°, 34.7°, 40.4°, and 42.9°, respectively, clearly indicating that the analyzed MAPbI_3_ film is of high phase purity. Because there is no controversy in the literature about the phase transition sequence of MAPbI_3_, which is as mentioned above, the low-temperature phase of MAPbI_3_ is classified as orthorhombic.

We now turn to the discussion of the main results of this work. To study the bandgap evolution, determine pressure coefficients, and examine their dependence on temperature, PL spectra of MAPbI_3_ films were recorded from ambient pressure to 6 GPa at three different temperatures within the respective OP (Figure 1a–c). All spectra were normalized to their maximal intensity and vertically shifted for ease of comparison. A high-energy peak is related to the orthorhombic matrix, whereas a low-energy peak is associated with small tetragonal domains.^12,34^ The tetragonal inclusions are not the subject of this work, and therefore, their pressure dependence is not discussed further. Pressure dependences of the energy positions of the OP peak were extracted from the PL spectra by Gaussian fit and are plotted in Figure 1d–f.

**Figure 1:**
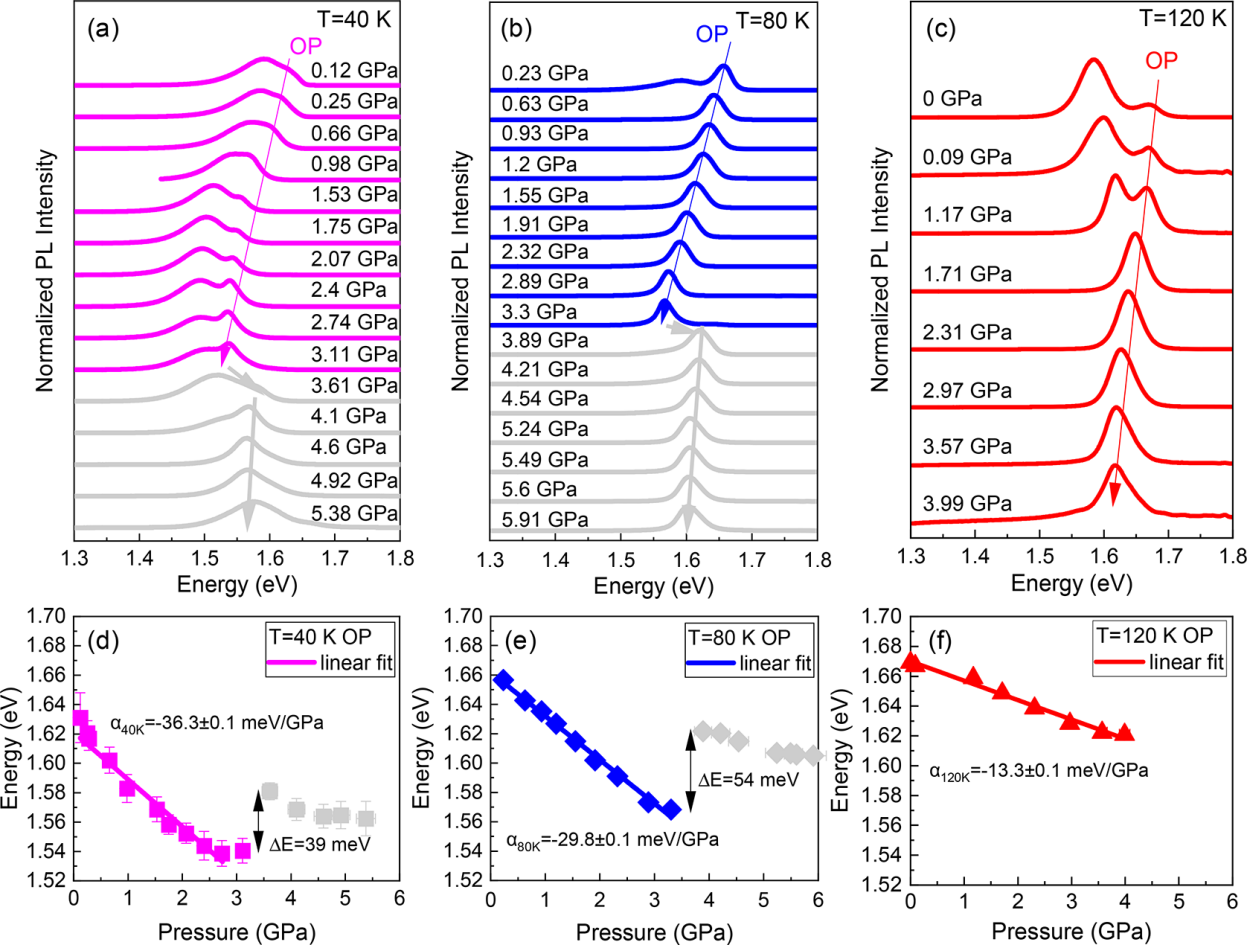
Normalized PL spectra of MAPbI_3_ as a function of pressure at (a) 40, (b) 80, and (c) 120 K. Peaks related to the orthorhombic phase (OP) are marked by arrows. The low-energy-side peak is ascribed to the tetragonal domains and not analyzed in this work. Colorful (gray) spectra were recorded in the pressure regime before (after) the blue-shift caused by lattice distortions. Pressure responses of the PL peak positions at (d) 40, (e) 80, and (f) 120 K. Solid lines indicate linear functions fitted to the pressure range, from which the pressure coefficient was determined. Where not indicated, error bars are smaller than the symbols.

According to previously performed DFT studies,^4,29,35^ pressure-induced Pb–I bond contraction leads to greater Pb 6p and I 5p orbital overlap, increased band dispersion, and a reduced bandgap, while rotations of [PbI_6_]^4–^ octahedra due to changes in the Pb–I–Pb bond angle suppress the Pb 6p and I 5p orbital overlap causing reduced band dispersion and consequently an increased bandgap. Our DFT results show that MAPbI_3_ crystal compression involves both shortening of the Pb–I bond (Figure 2a) and tilting of [PbI_6_]^4–^ octahedra (Figure 2b). Because the qualitative agreement between the experimental PL characteristics and the theoretical predictions of the bandgap evolution is good (discussed below), we can expect the trends of the structural factors derived from DFT to be meaningful. Figure 2a shows that as the pressure increases, the length of the Pb–I bonds decreases, which means that the pressure shortens the distance between the Pb and I atoms and leads to shrinkage of [PbI_6_]^4–^ octahedra. Below ∼5 GPa, the compressibility of the Pb–I bond is isotropic, and under higher pressures, it becomes clearly anisotropic; i.e., the Pb–I bond hardly shrinks within the *a–b* plane, while the shrinkage in the *a–c* plane is much larger. The pressure dependence of Pb–I–Pb angles (Figure 2b) also shows that the interoctahedral distortions increase with pressure. The octahedral tilting seems to be isotropic throughout the pressure range, as evidenced by the changes of the Pb–I–Pb angles within the *a–b* and *a–c* planes. [The Pb–I–Pb angle within the *a–b* (*a–c*) plane decreases from 159.9° (147.5°) at 0 GPa to 149.2° (137.7°) at 7.5 GPa.] Thus, we observe the increasing amplitude of pressure-induced intra- and interoctahedral distortions, which, as mentioned above, strongly influences the extent of the atomic orbital overlap and consequently the bandgap evolution under pressure.

**Figure 2:**
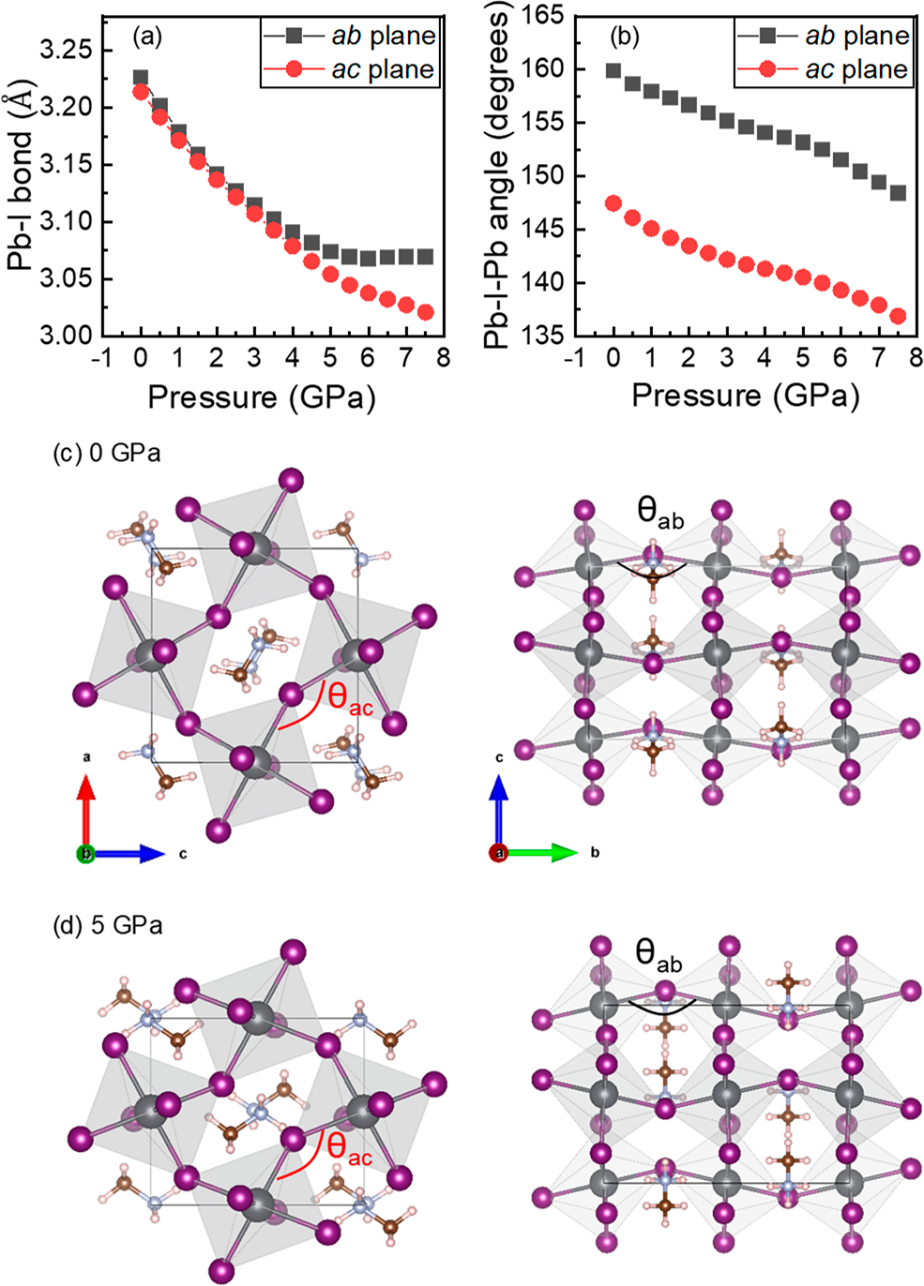
DFT results at zero temperature depicting (a) the compressibility of Pb–I bonds and (b) the pressure dependence of Pb–I–Pb angles. Fragments of lattices under (c) ambient and (d) high pressure in their *a–c* (left panels) and *b–c* (right panels) planes. Θ_ac_ and Θ_ab_ stand for Pb–I–Pb angles in the *a–c* and *a–b* planes, respectively.

In the case of the pressure response of the PL peak energy at 40 K (Figure 1a,d), comparison of experimental results with theoretical ones (Figure 2a,b) indicates that the red-shift observed during the compression from ambient pressure to 3.11 GPa is the outcome of two competitive effects, and in this pressure regime, Pb–I bond shrinkage has a stronger effect on the electronic structure than octahedral tilting. Simultaneously, PL spectra show a gradual increase in intensity (results shown in Figure S6a), which implies that along with increased orbital overlap, spectral absorption is extended and radiative recombination efficiency increases. At pressures between 3.11 and 3.61 GPa, the PL energy sharply blue-shifts due to decreased orbital overlap caused by a tilting of [PbI_6_]^4–^ octahedron relative to adjacent octahedra and consequently a distortion of the inorganic framework within the *a–c* and *a–b* planes (Figure 2c,d). We note that our DFT results do not show a sharp bend of the Pb–I–Pb angle under the transition pressure previously observed in the room-temperature single-crystal X-ray diffraction studies;^23,36^ nonetheless, inspection of panels a and b of Figure 2 reveals that at ∼5 GPa the pressure dependence of the Pb–I distance flattens anisotropically within the *a–b* and *a–c* planes, while continuous changes of the Pb–I–Pb angle become steeper, suggesting deviation from the preceding octahedral symmetry. A mentioned discrepancy in the measured and calculated transition pressures emerges from different temperatures of these studies and is discussed below. Further compression from 3.61 to 5.38 GPa causes more red-shifting of the peak position. This behavior also stems from the competing effects of Pb–I bond contraction and Pb–I–Pb angle bending. It is also worth mentioning that due to the reduced inorganic cage void volume, the MA cations become locked inside the voids and well-oriented along certain crystalline directions (Figure 2c,d), which locally increases the distortion of the inorganic octahedra.^26^ Further pressure increases to 5.97 GPa cause the disappearance of the PL emission (Figure S6b). Luminescence quenching is associated with the destabilization of the crystal structure due to significant distortion of [PbI_6_]^4–^ octahedra. It is important to note that all of the changes in the PL emission are reversible, although there is a certain degree of hysteresis. The PL peak recovers at 5.24 GPa during decompression (results shown in Figure S6a). Previously, it was reported for room-temperature studies that after pressurization of MAPbI_3_ to the peak pressure (the highest applied pressure) of 3.5 GPa, the PL band recovered as the pressure was gradually released at 1.7 GPa. However, after application of a pressure of 6.5 GPa, the PL peak did not recover.^25^ This is taken as evidence of better preservation of the inorganic framework at low temperatures, probably due to a reduced population of phonon modes because the transverse acoustic phonons can easily displace the iodide anion from the Pb–Pb midpoint and thus distort [PbI_6_]^4–^ octahedra.^37^ A situation similar to that at 40 K is observed for the PL pressure response measured at 80 K (Figure 1b,e). The trend of the peak positions changes at a pressure of 3.89 GPa, and the PL peak disappears at 5.99 GPa. It is also worth noting that the blue-shift caused by octahedral tilting is larger at 80 K (54 meV) than at 40 K (39 meV), which supports the effect of the temperature on the magnitude of octahedral distortion. The observed behavior of MAPbI_3_ thin films under pressure at 40 and 80 K is similar to that of single crystals compressed at room temperature with the initial red-shift of the PL energy positions followed by a blue-shift.^19,20,26^ Similar behavior has been also observed in room-temperature pressure studies of other halide perovskites and can be regarded as an indicator of the phase transition occurring in the studied pressure range.^38−40^ In our case, the observed blue-shift may also be a phase transition marker, but phase transition studies require structural confirmation and are not the aim of this work. At 120 K, the bandgap red-shift is observed up to 3.99 GPa (Figure 1c,f), while the bandgap blue-shift does not occur in the studied pressure range. The PL peak weakens substantially beyond 2.31 GPa and vanishes under a peak pressure of 4.12 GPa (results shown in Figure S7a,b), which is attributed to the atomic distortion-induced process. The luminescence quenching is reversible. The PL emission recovers at 3.75 GPa after the pressure is released (results presented in Figure S7a).

First-principles calculations within DFT (Figure 3) confirmed that the pressure-induced shifts in PL energy originate from changes in the fundamental bandgap. It is clear that the external hydrostatic pressure influences the electronic band structure. As shown in Figure 3a (black and green points), the bandgap narrows as the material is compressed from ambient pressure to ∼5 GPa as a consequence of the downshift of the bottom of the conduction band composed by the antibonding Pb 6p orbitals and the upshift of the valence band formed by antibonding Pb 6s orbitals hybridized with I 5p orbitals. Antibonding p orbitals show negative deformation potential as compared with antibonding s states,^41^ so the observed red-shift is at odds with the pressure dependence of the conventional semiconductors, in which the antibonding s states of the conduction band exhibit the blue-shift with pressure and bonding p-type orbitals of the valence band are almost insensitive to pressure.^41^ A blue-shift of the calculated bandgap is observed in the pressure range from 5 to 7 GPa as a result of the decreased overlap between the Pb 6p orbitals of the conduction band and the I 5p orbitals of the valence band. Then at ∼7 GPa a second red-shift occurs. Figure 3b shows the bandgap evolution under pressure for three selected values: 0, 5, and 7.5 GPa. According to the DFT calculations, the PL emission results from the direct optical transition at the Γ point of the Brillouin zone in the pressure range from ambient pressure to 7 GPa. At 7 GPa, the character of the bandgap starts changing from direct to indirect due to the upshift of the top of the valence band outside of the Γ toward the Z point, which can be responsible along with reduced orbital overlap for luminescence quenching at ∼6 GPa at 40 and 80 K. The detailed results of the described phenomenon obtained from the DFT calculations are provided in Figure S8. From 7 GPa onward, the optical bandgap (Figure 3a, black triangles) differs from the fundamental one (green stars). Comparison of the theoretical predictions (black and green points) with experimental results (red, blue, and magenta points) shows that the overall experimental behavior is captured well in these calculations; i.e., the red-shift–blue-shift–red-shift trend is retained [except for results obtained at 120 K (red triangles), where only a red-shift is observed in the considered pressure range]. However, quantitatively the agreement is not ideal. The difference becomes more significant with an increase in temperature, which is expected because the phonon contribution excluded in the DFT is enhanced with temperature. Tilting distortion of [PbI_6_]^4–^ octahedra is responsible for the observed blue-shift due to the reduction of orbital overlap and, consequently, the transition from the direct to indirect bandgap. We believe that the mentioned tilting distortion is strongly affected by acoustic phonon modes. In general, low-frequency acoustic phonon modes induce atomic displacement corresponding to macroscopic crystal deformation and affect the bandgap via either the deformation potential or a piezoelectrically induced electric field.^42,43^

**Figure 3:**
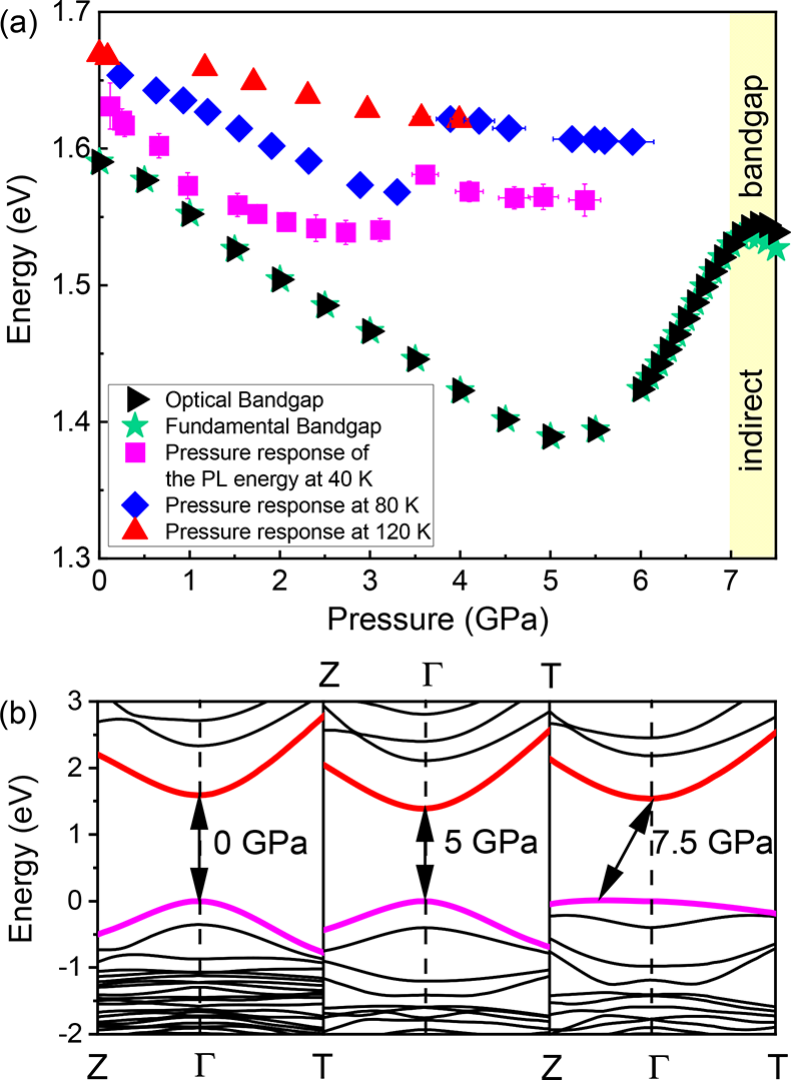
(a) Bandgap of MAPbI_3_ as a function of pressure obtained at 40 K (magenta squares), 80 K (blue diamonds), and 120 K (red triangles) together with the DFT results for optical (black triangles) and fundamental (green stars) bandgaps at zero temperature. The yellow area marks the pressure regime in which an indirect bandgap is a fundamental one. (b) Representative band structure under at 0 GPa (left), 5 GPa (middle), and 7.5 GPa (right), with the highest conduction and lowest valence bands colored red and magenta, respectively. Arrows indicate the fundamental bandgap. The energy scale is set with respect to the highest occupied state. Where not indicated, error bars are smaller than the symbols.

DFT is well-known to underestimate the bandgap of semiconductors, which results from the fact that the accuracy of this method depends on the employed functional and the selection of other parameters.^44−46^ Although the energies of direct optical transition can be underestimated, we can expect that their relative changes associated with the pressure response of the atomic system should give more reliable values because the errors related to different temperatures of experimental data and theoretical calculations, exciton binding energy, phonon contribution, and other inaccuracies in the DFT should be minimized. The pressure coefficient describes the evolution of the band structure, and therefore, it is reasonable to analyze only relative changes in the PL energies. Figure 4 shows the relative changes of the PL peak positions within the red-shift range of the pressure response with respect to the PL energy at ambient pressure (colorful points and lines) together with the DFT (black triangles and black lines). The PL energies at 0 GPa were determined by extrapolation of linear functions fitted in Figure 1d–f to the low-pressure range results (lines of the corresponding colors). The agreement between experimental data and theoretical predictions is best in the case of the lowest temperature, i.e., 40 K. With an increase in temperature, the difference also increases, which confirms the significant contribution of phonon modes in MAPbI_3_ with strong electron–phonon coupling.^47^ The inset of Figure 4 illustrates the temperature dependence of the pressure coefficient value within the OP. The experimental pressure coefficient determined in the pressure range from 0 to ∼3 GPa, i.e., the red-shift range in Figure 1d–f, has the following values: α_120K_ = −13.3 ± 0.1 meV/GPa, α_80K_ = −29.8 ± 0.1 meV/GPa, and α_40K_ = −36.3 ± 0.1 meV/GPa. The pressure coefficient calculated theoretically within the DFT framework at zero temperature is −41 meV/GPa. The absolute values of the pressure coefficient decrease with an increase in temperature. Such behavior can be explained by the fact that the red-shift of the bandgap associated with Pb–I bond contraction is partially suppressed by blue-shift caused by octahedral distortion, the magnitude of which is influenced by phonon modes.

**Figure 4:**
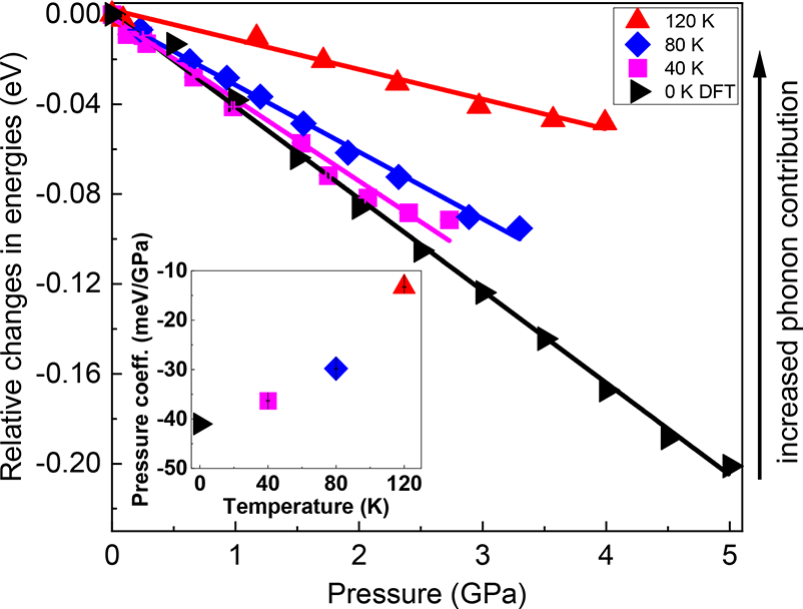
Relative changes of the PL energies within the orthorhombic phase (OP) at 40 K (magenta squares), 80 K (blue diamonds), and 120 K (red triangles) together with their linear fits (solid lines of the corresponding color) and results of theoretical calculations at zero temperature (black triangles and fitted solid black line). The linear pressure response of the relative PL energies is clearly visible. The inset shows the dependence of the pressure coefficient on the temperature in the OP. Error bars are smaller than the plotted symbols.

In the case of conventional semiconductors, which do not undergo a temperature-induced phase transition, pressure coefficients have been found to be almost independent of temperature.^48,49^ On the contrary, the pressure coefficients of materials with temperature-driven phase transitions, e.g., silicon, exhibit temperature dependence.^50,51^ We believe that in the case of MAPbI_3_, the structural factor playing a role in the dependence of the pressure coefficient on temperature is the increase in the Pb–I bond length and the unit cell volume with the symmetry reduction,^35,52,30^ which results in the better stability of [PbI_6_]^4–^ octahedra against pressure-induced tilting in a lower-temperature regime. This is supported by the fact that an abrupt blue-shift of the PL energy positions prompted by octahedral tilting occurs in the OP only after bond contraction at a pressure slightly below 4 GPa (Figure 1), and according to refs (19−21), the TP reveals tilt rotations under a significantly lower pressure of ∼0.3 GPa. Moreover, the cubic phase with a considerably shorter Pb–I bond length exhibits a positive pressure coefficient as the PL peak positions blue-shift with an increase in pressure over the whole pressure range, probably due to immediate significant octahedral tilting (results presented in Figure S9a,b). Therefore, octahedral tilting, which suppresses the effect of bond shrinkage on the pressure response of the bandgap of MAPbI_3_, is affected by not only the phonon population but also the initial Pb–I bond length. Thus, this leads to a decrease in the absolute values of the pressure coefficient with an increase in temperature.

In summary, this work shows experimental and theoretical studies of the pressure-induced evolution of the electronic structure of the OP of MAPbI_3_. We have shown that the bandgap of the MAPbI_3_ in OP can be tuned in a broader range of pressure (≲3.5 GPa) when compared to the TP, in which a red-shift of PL peak is observed only up to ∼0.3 GPa.^19−21^ Such behavior results from the better stability of the crystal lattice against octahedral distortions in the low-temperature regime due to the reduced phonon contribution and different Pb–I bond geometry. We found the pressure coefficient of the OP to be negative and dependent on the temperature. A negative deformation potential results from the antibonding character of the bottom of the conduction band and the top of the valence band, while dependence on the temperature can be explained by changes in the unit cell volume and geometry as the atomic configuration approaches the temperature-induced phase transition as well as phonon contribution. On the whole, our results advance the understanding of the behavior of the low-temperature MAPbI_3_ phase under extreme conditions and show that hydrostatic pressure allows fine-tuning of structural and optical properties, resulting in the possibility of realizing materials with the desired properties for technological applications.

## Supporting Information Available

Results supporting analysis of temperature-dependent PL presented in Note 1, including temperature-dependent PL spectra (Figure S1a), the dependence of the PL energy positions (Figure S1b) and full width at half-maximum (Figure S1c) on temperature, the intensity ratio of the OP emission and the TP emission at different temperatures within the respective OP (Figure S2), a scanning electron microscope image of a MAPbI_3_ film (Figure S3), low-temperature (≤120 K) PL results (Figures S4, S6, and S7), a powder XRD pattern at room temperature (Figure S5), computational details (Figure S8), the pressure response at 350 K (Figure S9), and Materials and Methods (PDF)
