# Supplementary material for: Bandgap Pressure Coefficient of a CH3NH3PbI3 Thin Film Perovskite
Source: J Phys Chem Lett. 2023 Jul 12;14(28):6470–6. doi: 10.1021/acs.jpclett.3c01258 (PMC10364135; doi:10.1021/acs.jpclett.3c01258)
Supplement: Supplementary file 1 — jz3c01258_si_001.pdf [file jz3c01258_si_001.pdf]

Supporting Information for

Bandgap Pressure Coefficient of $CH_3NH_3PbI_3$ Thin Film Perovskite

Agnieszka Pieniążek,[1] Filip Dybała,[1] Maciej P. Polak,[2] Łukasz Przypis,[1,3] Artur P. Herman,[1] Jan Kopaczek,[1] Robert Kudrawiec[1]*
[1]Department of Semiconductor Materials Engineering, Wroclaw University of Science and Technology, Wybrzeże Wyspiańskiego 27, 50-370 Wrocław, Poland
[2]Materials Science and Engineering Department, University of Wisconsin-Madison, Madison, WI 53706, USA
[3]Saule Research Institute, Wroclaw Technology Park, 11 Dunska Street, Sigma Building, 54-130 Wrocław, Poland

[*] to address correspondence:
agnieszka.pieniazek@pwr.edu.pl

Note 1 of Supporting Information: Analysis of temperature-dependent PL studies:

Figure S1a displays the PL spectra of a representative 250 nm thick $MAPbI_3$ film collected upon cooling in a temperature range going from 300 to 20 K with an interval of 20 K. The trend of the PL energy position (Fig. S1b) and the full-width at half maximum (Fig. S1c) can be used to indicate the phase transition point. We find that the onset of the transition from the TP to the OP during the cooling cycle is between 160 and 140 K, so the pressure response of the OP was investigated at temperatures below 140 K. Within the respective TP, the emission peak exhibits a redshift (Fig. S1b) and the full-width at half maximum (FWHM) narrows (Fig. S1c) with the decreasing temperature. The redshift of the emission peak is expected since the top of the valence band and the bottom of the conduction band have strong antibonding character.[1] The reducing FWHM with the decreasing temperature is ascribed to weaker electron-phonon interaction at lower temperature. At 140 K the emergence of the weak peak blue-shifted by about 100 meV with respect to the lower energy peak is observed (Fig. 1a). Further decreasing the temperature to 40 K, two emissions are clearly visible and the intensity ratio of the higher energy peak to the lower energy peak increases (Fig. S2). At 20 K the spectral positions of two emissions are close and peaks merge together. Within the OP (≤140 K) the higher energy peak is ascribed to the OP, whereas the lower energy emission originates from the tetragonal domains within orthorhombic matrix[2–7], which is consistent with theoretical calculations that the OP has wider bandgap than that of the TP[8]. This is argued to be caused by the polycrystalline nature of the sample, where grains can have a slightly different transition temperature due to, e.g., the different grain sizes. Scanning electron microscope images provided in Figure S3 show that the distribution of morphological grain sizes is quite large. Note that PL emphasizes even small fraction of tetragonal phase due to carrier transfer from orthorhombic to tetragonal phase evoked by internal electric field. Emission peak related to the OP is redshifted with the decreasing temperature (Fig. S1b) due to Pb-I bond contraction and shows the reducing FWHM (Fig. S1c) as a consequence of temperature-induced weakening of electron-phonon interactions similarly to the peak behaviour observed within the respective TP. Visual inspection of the graph in Figure S1c shows a non-zero gradient of the FWHM versus temperature in the low-temperature regime indicating considerable phonon contribution. Homogeneous broadening of

the PL peak in the low-temperature regime is expected to result predominantly from low-frequency acoustic phonons. Although in the case of polar $MAPbI_3$ energy of the longitudinal optical (LO) phonons is around 11.5 meV,[9] so their population at low temperature should not be very small. Also exponential shape of the temperature dependence of FWHM of the OP peak suggests LO phonon contribution in the linewidth. A noticeable blueshift of the peak originating from tetragonal inclusions in orthorhombic matrix (Fig. S1b) is probably due to the increasing quantum confinement effect in the small tetragonal domains as their size decreases with the decreasing temperature. The quantum confinement effect is likely to be the main factor responsible for the observed blueshift when size of the tetragonal domains is close to the bulk exciton Bohr radius (2.2 nm).[10] The FWHM of tetragonal domains increases with decreasing temperature (Fig. S1c), which might be caused by their size variation.

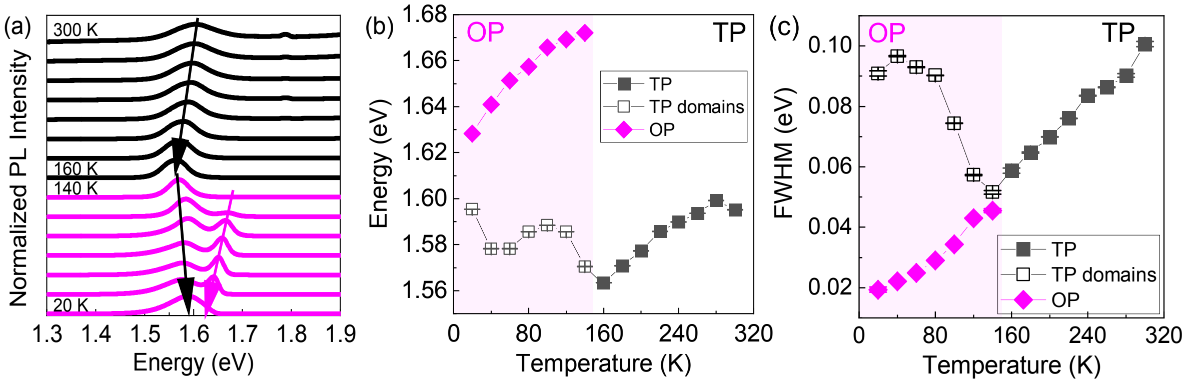

Figure S1. a) Temperature-dependent PL spectra of 250 nm thick $MAPbI_3$ film under ambient pressure. Arrows indicate the temperature cycle from 300 to 20 K with a step of 20 K. (b) and (c) Dependence of the PL energy positions and FWHM on the temperature, respectively. OP and TP stand for orthorhombic and tetragonal phases, respectively. The pink area marks the temperature range of the respective OP. Where not indicated, error bars are smaller than the symbols.

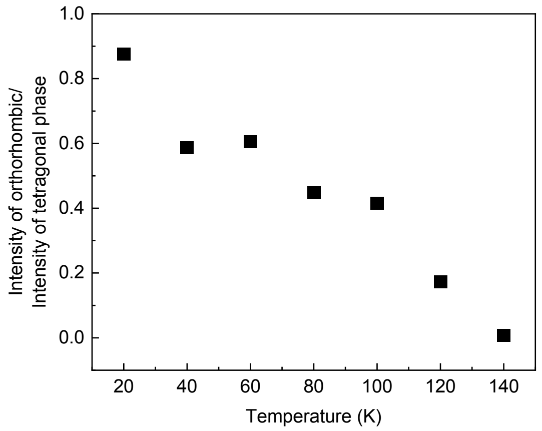

Figure S2. The photoluminescence (PL) intensity ratio of the higher energy (orthorhombic) peak to the lower energy (tetragonal) peak.

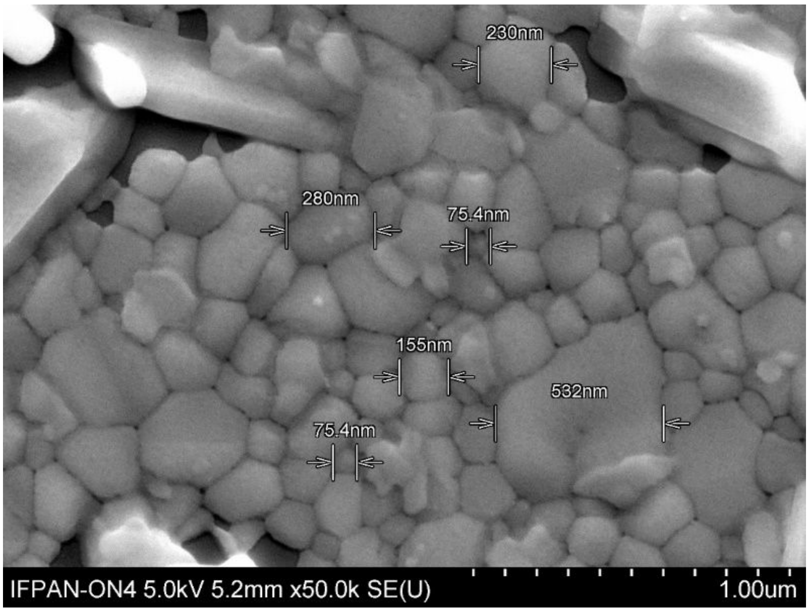

Figure S3. Scanning electron microscope image of $MAPbI_3$ film.

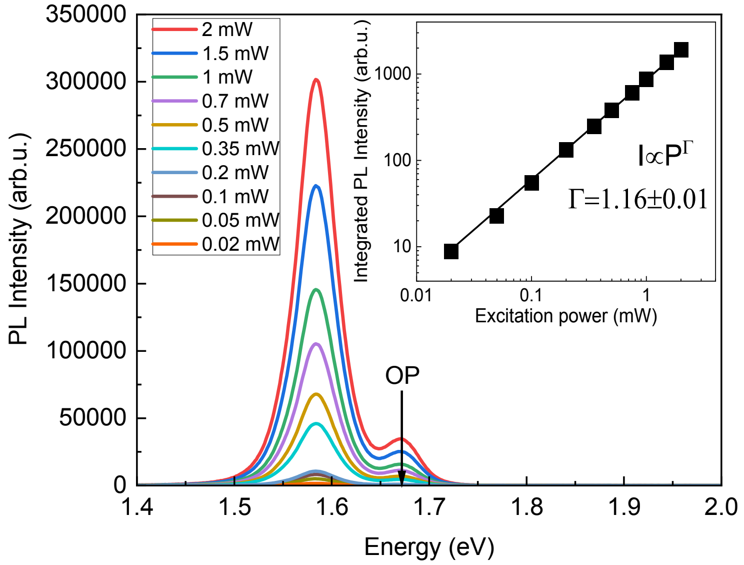

Figure S4. Excitation power-dependent PL spectra of $MAPbI_3$ film collected at 120 K under ambient pressure. The low-energy side emission originates from small tetragonal domains, the high-energy side emission comes from orthorhombic matrix. Inset shows the power law function fitted to the dependence of the integrated PL intensity on the excitation power. Error bars are smaller than the plotted symbols.

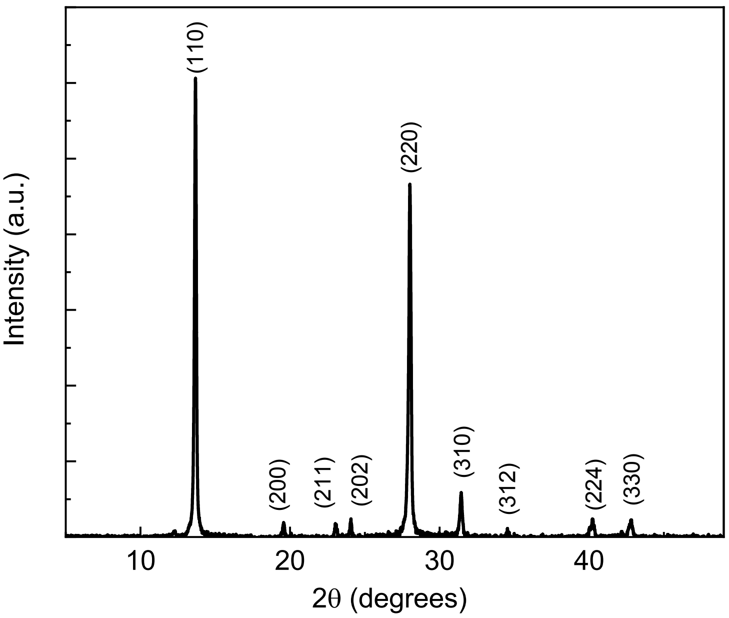

Figure S5. Powder XRD pattern of $MAPbI_3$ film at room temperature.

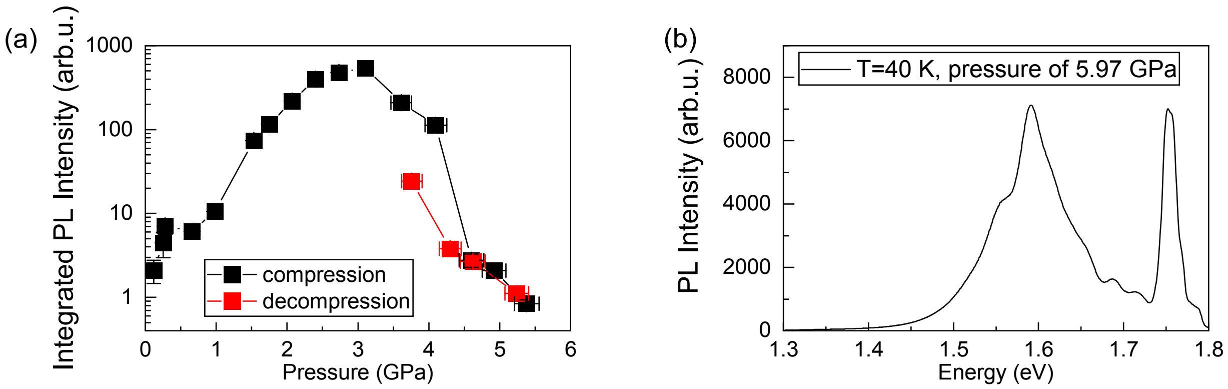

Figure S6. a) Dependence of the integrated PL intensity at 40 K during compression (black squares) and decompression (red squares), b) PL spectrum of ruby after PL of the sample vanished collected at 5.97 GPa. Where not indicated, error bars are smaller than the symbols.

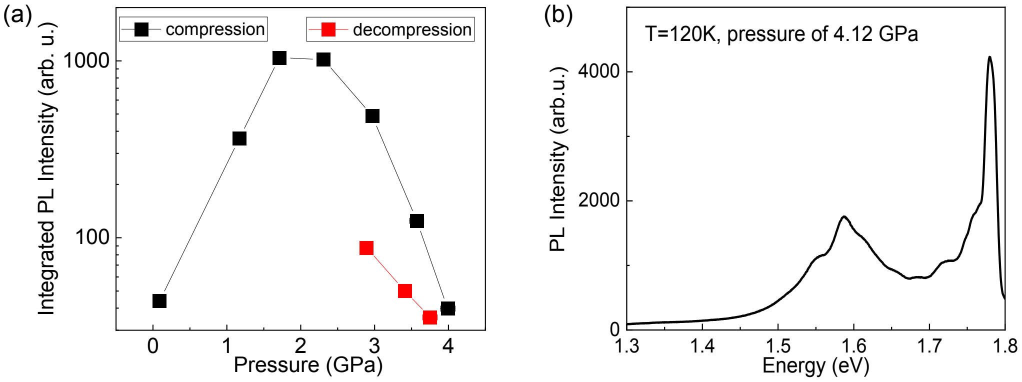

Figure S7. Dependence of a) the integrated PL intensity on pressure at 120 K during compression (black squares) and decompression (red squares), b) PL spectrum of ruby after PL of the sample was quenched collected at 4.12 GPa. Where not indicated, error bars are smaller than the symbols.

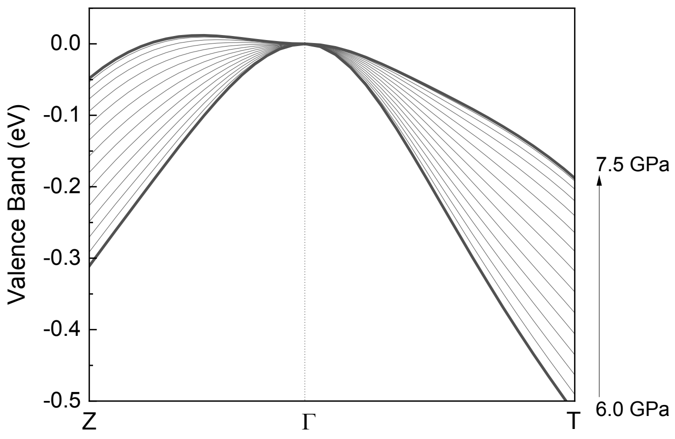

Figure S8. DFT calculations at zero temperature showing changes in the character of the bandgap under applied pressure.

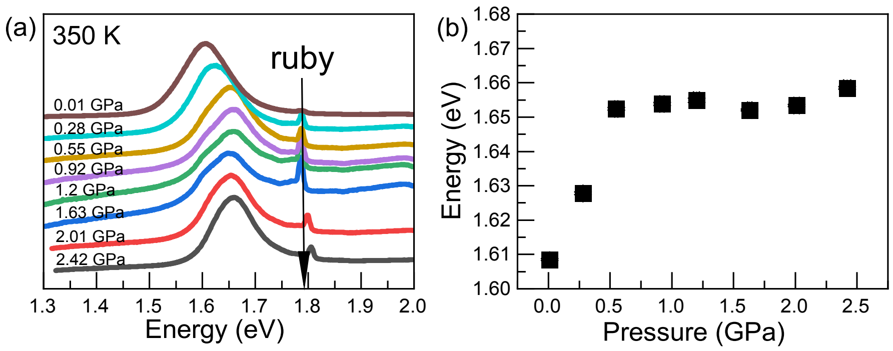

Figure S9. a) Pressure-dependent PL spectra of 250 nm thick $MAPbI_3$ film at 350 K. b) Pressure response of the PL peak positions at 350 K. Where not indicated, error bars are smaller than the symbols.

MATERIALS AND METHODS

Sample Preparation.

The $CH_3NH_3PbI_3$ perovskite solution was prepared by dissolving powders of MAI (159 mg) and PbI_2 (461 mg) in 1 ml of 9:1 (v/v) DMF/DMSO. Glass substrates were sonicated sequentially in deionized water, acetone and isopropanol, then dried with nitrogen and treated with oxygen plasma for 30 seconds to remove organic residues. Immediately thereafter, the substrates were transferred to a nitrogen-filled glovebox (H_2O<0.1 ppm; O_2<0.1 ppm). Since the perovskite precursor solution has poor wetting due to the surface energy of the substrate, the next rewetting step was performed using a UV lamp for 10 minutes. The perovskite layer was deposited by a two-step spin-coating procedure using toluene as an antisolvent. To remove the remaining solvents, the sample was transferred to a hotplate and annealed for 10 minutes at 100 °C. Next, the top perovskite layer was covered with a 2 μm PMMA layer. A PMMA solution in anisole was deposited by a one-step spin-coating procedure. The sample was then annealed on the hotplate (20 min at 60 °C) to remove the residues. For pressure studies, the perovskite stacks along with PMMA were peeled off from the original substrate.

Characterizations Perovskite Layer on Glass.

Film thicknesses were determined using a surface profilometer (Veeco Dektak 150). In all cases, the perovskite film thickness was ~250 nm.

Temperature and Pressure Dependent PL.

High-pressure PL measurements were carried out in Diacell design diamond anvil cells (DAC) produced by Almax easyLab. The cell body of DAC is made of beryllium copper (BeCu), which is suitable for cryogenic applications. We used diamonds with 650 μm culet size and additional single bevel facet made at 8 deg to enhance their mechanical resistance. The DAC was driven using gas membrane with control by adjusting the helium pressure. Daphne 7474 (a type of silicon oil) was used as the pressure transmitting medium ensuring hydrostatic conditions within the studied pressure range. Daphne 7474 solidifies at 3.7 GPa at room temperature, but despite this property, the transverse pressure distribution remains uniform up to 10 GPa over a wide range of 100 μm from the culet center.[11] The BeCu gasket was preindented from the initial thickness of 250 μm to a thickness of 50 μm. A central hole of about 1/3 culet size was made using traditional mechanical drilling. The pressure inside DAC were determined by pressure-induced redshift of ruby sphere R1 PL line with calibration performed by Shen *et al.*.[12] The low temperature was achieved through a two-stage cryostat with a closed loop liquid helium system. Temperature shift correction of the R1 ruby line was taken from the work of Datchi *et al.*.[13] All spectra were recorded using a 0.5 m Andor monochromator with a 150 g/mm diffraction grating blazed at 500 nm and a Si CCD camera cooled down to -70°C by Peltier elements. A 532 nm laser was used to excite the luminescence from the sample as well as to excite the R1 luminescence of the ruby.

Theoretical calculations.

First principles calculations were carried out within the DFT with the use of Vienna *ab initio* Simulation Package (VASP) code[14–16] and projector augmented-wave (PAW) potentials[17]. The valence orbitals of the used potentials were $5d^{10}6s^26p^2$, $5s^25p^5$, $2s^22p^2$, $2s^22p^3$, for Pb, I, C, and N, respectively. It has been shown that van der Waals interactions play an important role in proper description of the geometry and atomic arrangement in halide perovskites,[18] therefore for geometry optimization the rev-vdW-DF2 functional was used.[19] Lattice vectors and atomic positions were optimized at target pressures ranging from 0 to 7.5 GPa until the total energy was converged within at least 10^{-5} eV/atom and maximum residual forces acting on atoms were not exceeding 0.001 eV/Å. A conjugate gradient algorithm was used for geometry optimization. The calculated equilibrium lattice parameters were a=8.8815 Å, b=12.7069 Å, and c=8.5604 Å, which compared to the experimental values[20] of a=8.8362 Å, b=12.5804 Å, and c=8.5551 Å present a quite good agreement, with errors not surpassing 1%. A 4×3×4 k-point mesh was used with the plane-wave basis set determined by the cut-off energy of 550 eV. The electronic band structure was then calculated on the preoptimized structures with the hybrid HSE06 functional.[21] This approach allowed to benefit from the high accuracy of the rev-vdW-DF2 functional in describing the structure of the system, as proven by the great agreement with experimental parameters, and from the good description of the band structure of the HSE06. A similar approach has been demonstrated to be successful in calculations of the band structures of perovskites in the past.[18] While using HSE06 for both the geometry optimization and the electronic band structure calculations would have been the most consistent approach, the high computational cost of HSE06 prevented us from using it for geometry optimization. Therefore, we have chosen to combine the structure optimized with rev-vdW-DF2 with HSE06 calculations of the band structure, even though the rev-vdW-DF2 structure does not correspond to the HSE06 minimum energy structure. Although the hybrid functionals significantly improve the description of the band gap, in the case of halide perovskites the bandgap is still slightly underestimated. In order to compensate for the underestimation, the α parameter was adjusted to α=0.35 to properly reproduce the bandgap at 0 K and under zero pressure. The α has been kept constant for calculations under pressure. Spin-orbit coupling effects were included in these calculations since they play a crucial role in halide perovskites.[22]

X-ray diffraction.
X-ray diffraction (XRD) pattern was collected using Rigaku MiniFlex600 diffractometer (Cu Kα radiation, λ=1.5406 Å).

References of Supporting Information:

(1) Yin, W. J.; Yang, J. H.; Kang, J.; Yan, Y.; Wei, S. H. Halide Perovskite Materials for Solar Cells: A Theoretical Review. *J. Mater. Chem. A* **2015**, *3*, 8926–8942.

(2) Galkowski, K.; Mitioglu, A. A.; Surrente, A.; Yang, Z.; Maude, D. K.; Kossacki, P.; Eperon, G. E.; Wang, J. T.; Snaith, H. J.; Plochocka, P.; Nicholas, R. J. Spatially Resolved Studies of the Phases and Morphology of Methylammonium and Formamidinium Lead Tri-Halide Perovskites. *Nanoscale* **2017**, *9*, 3222–3230.

(3) Li, D.; Wang, G.; Cheng, H.-C.; Chen, C.-Y.; Wu, H.; Liu, Y.; Huang, Y.; Duan, X. Size-Dependent Phase Transition in Methylammonium Lead Iodide Perovskite Microplate Crystals. *Nat. Commun.* **2016**, *7*, 11330.

(4) Liu, Y.; Lu, H.; Niu, J.; Zhang, H.; Lou, S.; Gao, C.; Zhan, Y.; Zhang, X.; Jin, Q.; Zheng, L. Temperature-Dependent Photoluminescence Spectra and Decay Dynamics of MAPbBr3 and MAPbI3 Thin Films. *AIP Adv.* **2018**, *8*, 095108.

(5) Shojaee, S. A.; Harriman, T. A.; Han, G. S.; Lee, J. K.; Lucca, D. A. Substrate Effects on Photoluminescence and Low Temperature Phase Transition of Methylammonium Lead Iodide Hybrid Perovskite Thin Films. *Appl. Phys. Lett.* **2017**, *111*, 023902.

(6) Stavrakas, C.; Zelewski, S. J.; Frohna, K.; Booker, E. P.; Galkowski, K.; Ji, K.; Ruggeri, E.; Mackowski, S.; Kudrawiec, R.; Plochocka, P.; Stranks, S. D. Influence of Grain Size on Phase Transitions in Halide Perovskite Films. *Adv. Energy Mater.* **2019**, *9*, 1901883.

(7) Poglitsch, A.; Weber, D. Dynamic Disorder in Methylammoniumtrihalogenoplumbates (II) Observed by Millimeter-Wave Spectroscopy. *J. Chem. Phys.* **1987**, *87*, 6373–6378.

(8) Even, J.; Pedesseau, L.; Katan, C. Analysis of Multivalley and Multibandgap Absorption and Enhancement of Free Carriers Related to Exciton Screening in Hybrid Perovskites. *J. Phys. Chem. C* **2014**, *118*, 11566–11572.

(9) Wright, A. D.; Verdi, C.; Milot, R. L.; Eperon, G. E.; Pérez-Osorio, M. A.; Snaith, H. J.; Giustino, F.; Johnston, M. B.; Herz, L. M. Electron-Phonon Coupling in Hybrid Lead Halide Perovskites. *Nat. Commun.* **2016**, *7*, 11755.

(10) Tanaka, K.; Takahashi, T.; Ban, T.; Kondo, T.; Uchida, K.; Miura, N. Comparative Study on the Excitons in Lead-Halide-Based Perovskite-Type Crystals CH3NH3PbBr3 CH3NH 3PbI3. *Solid State Commun.* **2003**, *127*, 619–623.

(11) Murata, K.; Yokogawa, K.; Yoshino, H.; Klotz, S.; Munsch, P.; Irizawa, A.; Nishiyama, M.; Iizuka, K.; Nanba, T.; Okada, T.; Shiraga, Y.; Aoyama, S. Pressure Transmitting Medium Daphne 7474 Solidifying at 3.7 GPa at Room Temperature. *Rev. Sci. Instrum.* **2008**, *79*, 085101.

(12) Shen, G.; Wang, Y.; Dewaele, A.; Wu, C.; Dayne, E.; Eggert, J.; Klotz, S.; Dziubek, K. F.; Loubeyre, P.; Fat, V.; Asimow, P. D.; Mashimo, T.; Wentzcovitch, R. M. M.; Shen, G.; Wang, Y.; Dewaele, A.; Wu, C.; Dayne, E.; Eggert, J.; Klotz, S.; Dziubek, K. F.; Loubeyre, P.; Fat, O. V; Asimow, D.; Mashimo, T.; Wentzcovitch, R. M. M. Toward an International Practical Pressure Scale: A Proposal for an IPPS Ruby Gauge (IPPS-Ruby2020). *High Press. Res.* **2020**, *40*, 299–314.

(13) Datchi, F.; Dewaele, A.; Loubeyre, P.; Letoullec, R.; Godec, Y. Le; Canny, B.; Dewaele, A.; Loubeyre, P.; Letoullec, R.; Godec, Y. Le; Canny, B. Optical Pressure Sensors for High-Pressure – High-Temperature Studies in a Diamond Anvil Cell. *High Press. Res.* **2007**, *27*, 447–463.

(14) Kresse, G.; Furthmiiller, J. Efficiency of Ab-Initio Total Energy Calculations for Metals and Semiconductors Using a Plane-Wave Basis Set. *Comput. Mater. Sci.* **1996**, *6*, 15–50.

(15) Kresse, G.; Furthmüller, J. Efficient Iterative Schemes for Ab Initio Total-Energy Calculations Using a Plane-Wave Basis Set. *Phys. Rev. B* **1996**, *54*, 11169–11186.

(16) Kresse, G.; Hafner, J. Ab Initio Molecular Dynamics for Liquid Metals. *Phys. Rev. B* **1993**, *47*, 558–561.

(17) Kresse, G.; Joubert, D. From Ultrasoft Pseudopotentials to the Projector Augmented-Wave Method. *Phys. Rev. B* **1999**, *59*, 1758–1775.

(18) Tao, S. X.; Cao, X.; Bobbert, P. A. Accurate and Efficient Band Gap Predictions of Metal Halide Perovskites Using the DFT-1/2 Method: GW Accuracy with DFT Expense. *Sci. Rep.* **2017**, *7*, 14386.

(19) Hamada, I. Van Der Waals Density Functional Made Accurate. *Phys. Rev. B* **2014**, *89*, 121103.

(20) Baikie, T.; Fang, Y.; Kadro, J. M.; Schreyer, M.; Wei, F.; Mhaisalkar, S. G.; Graetzel, M.; White, T. J. Synthesis and Crystal Chemistry of the Hybrid Perovskite (CH3NH3)PbI3 for Solid-State Sensitised Solar Cell Applications. *J. Mater. Chem. A* **2013**, *1*, 5628–5641.

(21) Krukau, A. V.; Vydrov, O. A.; Artur F. Izmaylov, et al. Influence of the Exchange Screening Parameter on the Performance of Screened Hybrid Functionals. *J. Chem. Phys.* **2006**, *125*, 224106.
(22) Even, J.; Pedesseau, L.; Jancu, J.-M.; Katan, C. Importance of Spin–Orbit Coupling in Hybrid Organic/Inorganic Perovskites for Photovoltaic Applications. *J. Phys. Chem. Lett.* **2013**, *4*, 2999–3005.
